# The second round of the Dutch colorectal cancer screening program: Impact of an increased fecal immunochemical test cut‐off level on yield of screening

**DOI:** 10.1002/ijc.32839

**Published:** 2020-01-09

**Authors:** Arthur I. Kooyker, Esther Toes‐Zoutendijk, Annemieke W.J. Opstal‐van Winden, Manon C.W. Spaander, Maaike Buskermolen, Hanneke J. van Vuuren, Ernst J. Kuipers, Folkert J. van Kemenade, Chris Ramakers, Maarten G.J. Thomeer, Evelien Dekker, Iris D. Nagtegaal, Harry J. de Koning, Monique E. van Leerdam, Iris Lansdorp‐Vogelaar

**Affiliations:** ^1^ Department of Public Health Erasmus University Medical Center Rotterdam The Netherlands; ^2^ Department of Gastroenterology and Hepatology Erasmus University Medical Center Rotterdam The Netherlands; ^3^ Department of Pathology Erasmus University Medical Center Rotterdam The Netherlands; ^4^ Department of Clinical Chemistry Erasmus University Medical Center Rotterdam The Netherlands; ^5^ Department of Radiology Erasmus University Medical Center Rotterdam The Netherlands; ^6^ Department of Gastroenterology and Hepatology Amsterdam University Medical Centers – Academic Medical Center Amsterdam The Netherlands; ^7^ Department of Pathology Radboud University Medical Center Nijmegen The Netherlands; ^8^ Department of Gastroenterology Netherlands Cancer Institute – Antoni van Leeuwenhoek Amsterdam The Netherlands

**Keywords:** colorectal cancer, colorectal cancer screening, fecal immunochemical test, colorectal neoplasia, colonoscopy

## Abstract

The Dutch colorectal cancer (CRC) screening program started in 2014, inviting the target population biennially to perform a fecal immunochemical test (FIT). We obtained prospectively collected data from the national screening information‐system to present the results of the second round (2016) and evaluate the impact of increasing the FIT cut‐off halfway through the first round from 15 to 47 μg Hb/g feces on outcomes in the second round. Second round screening was done with a 47 μg Hb/g feces FIT cut‐off. Participants were classified based on first round participation status as either FIT (15,47) or FIT (47,47) participants, and previous nonparticipants. In total, 348,891 (75.9%) out of 459,740 invitees participated in the second round. Participation rates were 93.4% among previous participants and 21.0% among previous non‐participants. FIT(47,47) participants had a significantly higher detection rate of AN (15.3 *vs.* 10.4 per 1,000 participants) compared to FIT(15,47) participants in the second round, while their cumulative detection rate of AN over two rounds was significantly lower (45.6 *vs.* 52.6 per 1,000 participants). Our results showed that participation in the Dutch CRC screening program was consistently high and that second round detection rates depended on the first round FIT cut‐off. The cumulative detection over two rounds was higher among FIT(15,47) participants. These findings suggest that a substantial part of, but not all the missed findings in the first round due to the increased FIT cut‐off were detected in the subsequent round.

## Abstract 2

**What's new?**

In 2014, the Netherlands implemented colorectal cancer (CRC) screening based on non‐invasive fecal immunochemical testing (FIT), which offers a practical approach for population‐based CRC detection. In the Dutch program's first round, to match local resources, FIT cut‐off was increased, resulting in reduced positivity rates and reduced colonoscopy referrals, at the cost of missing advanced neoplasias. The current study shows that many of these missed advanced neoplasias were detected in subsequent screening, suggesting that increased FIT cut‐off had marginal impact on screening outcome. The findings could benefit other CRC screening programs in establishing effective FIT cut‐offs.

**Abbreviations**
AA: advanced adenoma
AN: advanced neoplasia
CRC: colorectal cancer
FIT: fecal immunochemical test
NNScope: number needed to scope
OR: odds ratio
PPV: positive predictive value
SRR: standardized rate ratio

## Introduction

Many countries have recently introduced colorectal cancer (CRC) screening with the aim to reduce CRC incidence and mortality. These programs use different screening strategies.^1^ Colonoscopy is the gold standard for detecting advanced neoplasia (AN) because of its high sensitivity. However, colonoscopy is an invasive procedure that demands extensive resources when used for primary screening on a population level. Many countries therefore prefer a non‐invasive fecal test for primary screening, followed by colonoscopy when tested positive. Of the currently available fecal tests, fecal immunochemical testing (FIT) is associated with the highest participation and a high diagnostic performance.^2^, ^3^, ^4^, ^5^, ^6^ Besides its superior characteristics compared to other fecal testing screen modalities, FIT offers the advantage to adjust the cut‐off level to match local resources.^7^ It allows for optimizing the balance between the number of true‐ and false‐positives, potentially influencing the detection rate.^8^, ^9^ Modeling studies based on real‐life data reported that annual FIT at a low cut‐off is equally effective in reducing CRC‐related mortality as 10‐yearly primary colonoscopy screening.^10^ However, many organized programs are currently forced to use a higher FIT cut‐off and a longer screening interval, often due to a limited colonoscopy capacity.^1^, ^11^, ^12^

The Dutch FIT‐based CRC screening program started in 2014 after extensive piloting in previous years. During the first months after the start, we observed a higher participation rate, a higher FIT positivity rate and a lower positive predictive value (PPV) compared to the results of the preceding pilot studies. Since the referral rate exceeded the colonoscopy capacity, the FIT cut‐off was increased halfway during the first year, resulting in a lower positivity rate and a higher PPV for AN.^13^ In this study we evaluated participation in the second round and estimated the impact of the adjusted FIT cut‐off in the first round, on screening outcomes in the second round. This information is relevant for screening programs worldwide when deciding on optimal implementation of FIT‐screening.

## Materials and Methods

The design of the Dutch CRC screening program and its real‐time monitoring system have previously been described.^13^ In summary, the target population consists of individuals aged 55–75 years old, who are invited every 2 years to perform a FIT (FOB‐Gold; Sentinel). The target population was invited gradually by birth‐cohort, with a projected rollout period of 5 years. Participants with a positive FIT (μg hemoglobin per gram of feces above the cut‐off) are referred for a pre‐colonoscopy intake. During this intake at the outpatient clinic, individuals are informed about the colonoscopy procedure and bowel preparation and assessed for eligibility. When considered eligible, individuals are scheduled for colonoscopy. In case of the detection of adenoma or CRC, the participant was referred for further treatment and colonoscopy surveillance.

### Study population

In 2014, at the start of the screening program, birth cohorts 1951, 1949, 1947, 1939 and 1938, that respectively reached the age of 63, 65, 67, 75 and 76 years, were invited for first‐round screening. These birth cohorts were selected according to a rollout strategy. We started in 2014 with the invitation of the oldest age groups because it was their only opportunity to participate. Persons aged 76 years were also included due to a delayed implementation of the program. For the second round, the same target group was re‐invited in 2016, except for the invitees who tested positive in the first round, who had become older than 75 years, or who had deregistered permanently from the screening program. In the first half year of 2014, a FIT cut‐off of 15 μg hemoglobin per gram (Hb/g) feces was used. This was increased to 47 μg in the second half of 2014. In the second round, all FIT samples were analyzed with a 47 μg FIT cut‐off.

### Data collection

Of all invitees of the first round, data on participation status, FIT‐result (μg Hb/g feces), pre‐colonoscopy intake and colonoscopy results in the first and/or second screening round were collected from the national screening information‐system (ScreenIT).

### Outcomes

Participation rate, FIT positivity rate, PPV for AN and detection rate of AN in the second round were the primary outcomes of this study. An invitee was considered a participant when a FIT stool‐sample was returned and a non‐participant when there was no response or when the invitee deregistered. The participation rate was defined as the number of participants divided by the number of individuals invited. The positivity rate was defined as the number of participants with a FIT‐result at or above the cut‐off divided by the number of participants with an assessable FIT. The participation rate for pre‐colonoscopy intake was defined as the number of persons who attended the intake divided by the number of FIT‐positives. The participation rate for colonoscopy was defined as the number of persons that underwent colonoscopy divided by the number of persons with a positive FIT. AN was considered a relevant finding within the CRC screening program and was defined as CRC or advanced adenoma (AA).^14^ AA was defined as any adenoma with histology showing 25% or greater villous component or high‐grade dysplasia or an adenoma with size of 10 mm or larger. The PPV for AN was calculated as the number of persons detected with AN divided by the number of persons who underwent a colonoscopy. The detection rate of AN was defined as the number of persons detected with AN of those who returned an assessable FIT.

Secondary outcomes were cumulative positivity rate, cumulative detection rate of AN, number needed to scope (NNScope) to detect AN in one patient, and the association between concentration μg Hb/g feces (FIT‐level) in the first round and screening‐outcomes in the second round. Cumulative positivity rate was defined as the number of positive FIT results over both rounds divided by the number of participants that returned an assessable FIT in both rounds or tested positive in the first round. The cumulative detection rate was defined as the number of CRC or AN detected over both rounds in participants that returned an assessable FIT in both rounds or tested positive in the first round. The NNScope was defined as the number of performed colonoscopies divided by the number of detected CRC or AN, over both rounds. The FIT‐level in the first round was tested on the association with positive FIT‐result, PPV and detection of AN in the second round.

### Analyses

All invitees to the second round were analyzed for participation rate, positivity rate, participation rate of pre‐colonoscopy intake and colonoscopy, PPV for AN and detection rate of AN. Primary outcomes were presented for three different subgroups: individuals that were tested with a FIT cut‐off of 15 μg Hb/g feces in the first round (FIT(15,47)), individuals that were tested with a FIT cut‐off of 47 μg Hb/g feces in the first round (FIT(47,47)) and individuals that did not participate in the first round (FIT(np,47)). Secondary outcomes excluded participants of the first round of the oldest birth cohorts (1938 and 1939), since those participants were not part of the target population of 2016 and therefore not re‐invited. The cumulative positivity rate, cumulative detection rate and NNScope over two screen‐rounds were compared between FIT(15,47) and FIT(47,47) participants of both rounds. To assess the association between FIT‐level in the first round and screening outcomes in the second round, the FIT‐results of first round participants were categorized in 0, 1–14 and 15–46 μg Hb/g feces (the latter group was only applicable to FIT(47,47) participants).

Descriptive statistics were computed of the primary outcomes, including 95% confidence intervals (95% CI). Differences between groups were tested for statistical significance (α < 0.05) using the Chi‐square test or the Student's *t*‐test. Age‐adjusted rates for the primary outcomes and secondary outcomes were calculated for groups with more than 50 invitees, therefore only age groups of 65, 67 and 69 years old were included. Because of substantially different age‐distributions between subgroups, the chi‐square was not applicable. Instead, differences between the age‐adjusted rates were tested with the standardized rate ratio (SRR).^15^, ^16^ If the 95% CI of the SRR includes 1, no significant difference was observed between the age‐adjusted rates.

Multivariable logistic regression was performed to estimate the odds ratios (ORs) of the FIT‐level in the first round on screening outcomes in the second round, adjusted for gender and age.

As sensitivity analyses, we (*i*) tested the outcomes on significant differences between gender and (*ii*) only included FIT(47,47) participants to rule out selection bias (Supporting Information Table S1 and S2). This bias could have occurred because FIT(15,47) participants were invited in the first half, and FIT(47,47) participants in the second half of 2014.

Data analyses were performed using R version 3.4.1.

### Ethical approval

The Dutch population screening program was approved by the Ministry of Health and the Dutch population screening act. According to the Central Committee on Research involving Human Subjects (CCMO), this study did not require approval from an ethics committee in the Netherlands. Returning the FIT is considered informed consent, in accordance with the Dutch population screening act. No identifying individual data were made available in this study.

## Results

**Figure 1:**
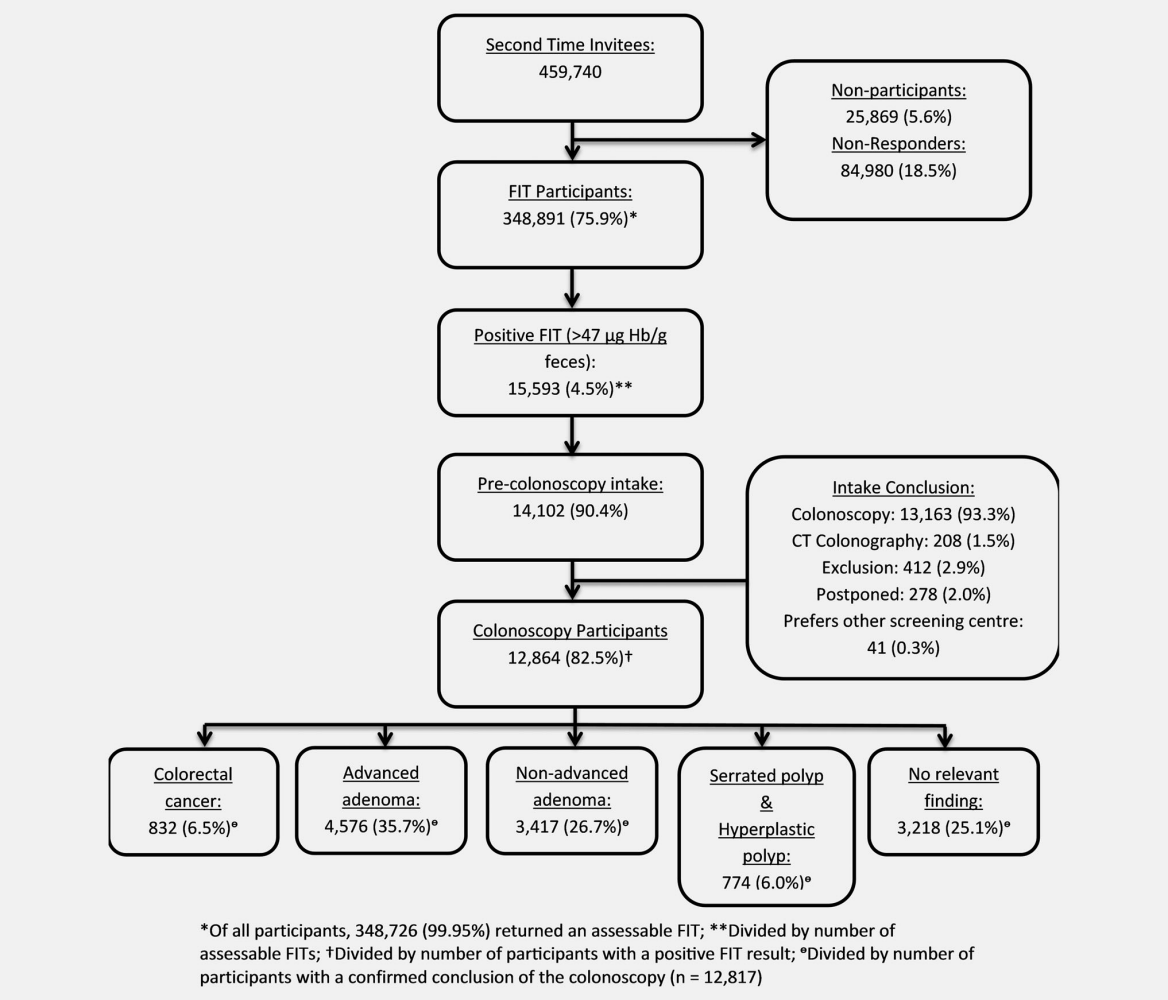
Flow chart of the second round of the Dutch colorectal cancer screening program.

In total, 459,740 individuals were invited for second‐round screening. Of those, 348,891 (75.9%) returned the FIT and 15,593 (4.5%) tested positive (Fig. 1 and Table 1). Out of the FIT‐positive participants, 14,102 (90.4%, 95% CI: 90.0–90.9%) individuals attended the pre‐colonoscopy intake, of which 13,163 (93.3%, 95% CI: 92.9–93.7%) were advised to undergo colonoscopy. Eventually, 12,864 (82.5%, 95% CI: 81.9–83.1%) participants of all individuals that tested FIT‐positive underwent colonoscopy, leading to detection of 832 CRCs and 4,576 AAs, resulting in a detection rate of 15.5 (95% CI: 15.1–15.9%) AN per 1,000 participants.

**Table 1:** Total yield of the second round

	Participation rate		Positivity rate		PPV CRC		PPV AA		Detection rate CRC		Detection rate AA	
	*n*	%	*n*	%	*n*	%	*n*	%	*n*	‰	*n*	‰
Total	348,891	75.9 (75.8–76.0)	15,593	4.5 (4.4–4.5)	832	6.5 (6.1–6.9)	4,576	35.7 (34.9–36.5)	832	2.4 (2.2–2.6)	4,576	13.1 (12.7–13.5)
Gender												
Men	168,916	74.0 (73.8–74.2)	8,998	5.3 (5.2–5.4)	495	6.7 (6.1–7.2)	2,878	38.7 (37.6–39.8)	495	2.9 (2.7–3.2)	2,878	17.0 (16.4–17.7)
Women	179,975	77.8 (77.6–77.9)	6,595	3.7 (3.6–3.8)	337	6.3 (5.6–6.9)	1,698	31.6 (30.3–32.8)	337	1.9 (1.7–2.1)	1,698	9.4 (9.0–9.9)
Age												
62	26,027	75.9 (72.4–73.3)	1,064	4.1 (3.9–4.3)	40	4.6 (3.4–6.2)	296	34.3 (31.2–37.5)	40	1.5 (1.1–2.1)	296	11.4 (10.2–12.7)
65	84,442	76.3 (76.0–76.5)	3,572	4.2 (4.1–4.4)	186	6.3 (5.5–7.2)	1,048	35.4 (33.7–37.1)	186	2.2 (1.9–2.5)	1,048	12.4 (11.7–13.2)
67	108,248	76.1 (75.8–76.3)	4,679	4.3 (4.2–4.4)	222	5.8 (5.1–6.5)	1,360	35.3 (33.8–36.8)	222	2.1 (1.8–2.3)	1,360	12.6 (11.9–13.3)
69	130,174	76.1 (75.9–76.3)	6,278	4.8 (4.7–4.9)	384	7.5 (6.8–8.2)	1,872	36.5 (35.2–37.8)	384	3.0 (2.7–3.3)	1,872	14.4 (13.8–15.0)

Note: Participants older than 75 years old (77 [*n* = 1] and 78 [*n* = 13]) are excluded from analyses. Because of small numbers, the age categories of 66 (*n* = 1) and 72 years old (*n* = 1) is not shown.

Abbreviations: AA, advanced adenoma; CRC, colorectal cancer; PPV, positive predictive value.

### Participation

Of all 348,071 second‐round invitees who participated in the first round, 325,392 (93.5%) also participated in the second round (Table 2). Of all 111,669 second‐round invitees who did not participate in the first round, a total of 23,499 (21.0%) participated in the second round.

**Table 2:** Outcomes of the second screening round in first round participants (tested with a low [15 μg Hb/g feces] or high [47 μg Hb/g feces] cut‐off level) and in first round non‐participants

	Participation rate		Positivity rate		PPV CRC		PPV AA		Detection rate CRC		Detection rate AA	
	*n*	%	*n*	%	*n*	%	*n*	%	*n*	‰	*n*	‰
Previous participants												
Total	325,392	93.5 (93.4–93.6)	13,743	4.2 (4.2–4.3)	708	6.1 (5.7–6.6)	3,996	34.6 (33.8–35.5)	708	2.2 (2.0–2.3)	3,996	12.3 (11.9–12.7)
Adjusted^1^		93.5		4.2		6.1		34.6		2.2		12.3
FIT (15,47)												
Total	39,257	94.1 (93.8–94.3)	1,303	3.3 (3.1–3.5)	63	5.7 (4.5–7.2)	344	31.2 (28.5–34.0)	63	1.6 (1.3–2.1)	344	8.8 (7.9–9.7)
Adjusted^1^				3.4^2^		5.5		30.5		1.6		8.8^2^
Gender												
Men	18,497	94.0 (93.7–94.4)	732	4.0 (3.7–4.2)	36	5.8 (4.2–7.9)	209	33.7 (30.0–37.5)	36	1.9 (1.4–2.7)	209	11.3 (9.9–12.9)
Women	20,760	94.0 (93.7–94.3)	571	2.8 (2.5–3.0)	27	5.6 (3.9–8.0)	135	28.0 (24.2–32.2)	27	1.3 (0.9–1.9)	135	6.5 (5.5–7.7)
Age												
65	1,953	94.5 (93.5–95.4)	67	3.4 (2.7–4.3)	2	3.6 (1.0–12.1)	15	26.8 (17.0–39.6)	2	1.0 (0.3–3.7)	15	7.7 (4.7–12.6)
67	23,874	94.0 (93.7–94.3)	741	3.1 (2.9–3.3)	32	5.1 (3.7–7.2)	191	30.7 (27.2–34.4)	32	1.3 (0.9–1.9)	191	8.0 (6.9–9.2)
69	13,430	94.1 (93.7–94.5)	495	3.7 (3.4–4.0)	29	6.8 (4.8–9.6)	138	32.5 (28.2–37.1)	29	2.2 (1.5–3.1)	138	10.3 (8.7–12.1)
FIT (47,47)												
Total	286,135	93.4 (93.3–93.5)	12,440	4.3 (4.3–4.4)	645	6.2 (5.7–6.7)	3,652	35.0 (34.1–35.9)	645	2.3 (2.1–2.4)	3,652	12.8 (12.4–13.2)
Adjusted^1^				4.4^2^		6.3		35.2		2.3		13.0^2^
Gender												
Men	138,117	93.4 (93.3–93.5)	7,108	5.1 (5.0–5.3)	373	6.2 (5.6–6.9)	2,269	37.9 (36.7–39.1)	373	2.7 (2.4–3.0)	2,269	16.4 (15.8–17.1)
Women	148,018	93.4 (93.2–93.5)	5,332	3.6 (3.5–3.7)	272	6.1 (5.5–6.9)	1,383	31.1 (29.8–32.5)	272	1.8 (1.6–2.1)	1,383	9.3 (8.9–9.8)
Age												
62	23,875	93.0 (92.7–93.3)	910	3.8 (3.6–4.1)	36	4.8 (3.5–6.5)	248	32.9 (29.7–36.4)	36	1.5 (1.1–2.1)	248	10.4 (9.2–11.8)
65	76,776	93.6 (93.4–93.8)	3,087	4.0 (3.9–4.2)	152	5.8 (5.0–6.8)	895	34.3 (32.5–36.2)	152	2.0 (1.7–2.3)	895	11.7 (10.9–12.4)
67	77,209	93.5 (93.3–93.6)	3,387	4.4 (4.2–4.5)	151	5.3 (4.5–6.2)	994	34.8 (33.1–36.6)	151	2.0 (1.7–2.3)	994	12.9 (12.1–13.7)
69	108,275	93.3 (93.2–93.5)	5,056	4.7 (4.5–7.8)	306	7.3 (6.5–8.1)	1,515	35.9 (34.5–37.4)	306	2.8 (2.5–3.2)	1,515	14.0 (13.3–14.7)
Previous non‐participants												
Total	23,499	21.0 (20.8–21.3)	1,850	7.9 (7.6–8.3)	124	9.7 (8.2–11.4)	580	45.2 (42.5–48.0)	124	5.3 (4.5–6.3)	580	24.8 (22.8–26.8)
Adjusted^1^		21.0^3^		7.9^3^		9.8^3^		45.3^3^		5.4^3^		24.8^3^
Gender												
Men	12,302	20.3 (19.9–20.6)	1,158	9.4 (8.9–10.0)	86	10.4 (8.5–12.7)	400	48.5 (45.1–52.0)	86	7.0 (5.7–8.7)	400	32.6 (29.6–35.9)
Women	11,197	22.0 (21.6–22.3)	692	6.2 (5.8–6.7)	38	8.3 (6.1–11.2)	180	39.3 (34.9–43.8)	38	3.4 (2.5–4.7)	180	16.1 (14.0–18.6)
Age												
62	2,152	21.4 (20.6–22.2)	154	7.2 (6.2–8.3)	4	3.6 (1.4–9.0)	48	43.6 (34.7–53.0)	4	1.9 (0.7–4.8)	48	22.4 (16.9–29.5)
65	5,713	21.5 (21.0–22.0)	418	7.3 (6.7–8.1)	32	10.7 (7.7–14.8)	138	46.3 (40.7–52.0)	32	5.6 (4.0–7.9)	138	24.2 (20.6–28.6)
67	7,165	20.9 (20.5–21.3)	551	7.7 (7.1–8.4)	39	10.2 (7.5–13.6)	175	45.7 (40.8–50.7)	39	5.5 (4.0–7.5)	175	24.5 (21.2–28.4)
69	8,469	20.8 (20.4–21.2)	727	8.6 (8.0–9.2)	49	10.0 (7.6–12.9)	219	44.6 (40.3–49.0)	49	5.8 (4.4–7.7)	219	25.9 (22.7–29.5)

Note: Because of small numbers, the results of the age categories of 66 (*n* = 1) and 72 (*n* = 1) years old in the non‐participants of the first round are not shown.

1 Age‐adjusted rates.

2 Significant (α = 0.05) difference between used FIT cut‐offs in the first round.

3 Significantly (α = 0.05) different compared to previous participants.

Abbreviations: AA, advanced adenoma; CRC, colorectal cancer; PPV, positive predictive value.

### Yield of screening

Among second‐round invitees that had also participated in the first round, there were 39,257 FIT(15,47) and 286,135 FIT(47,47) participants (Table 2). FIT(15,47) participants in the second round had a positivity rate of 3.3%, a PPV for AN of 36.9% (95% CI: 34.1–39.8%), and a detection rate of AN of 10.4 (95% CI: 9.4–11.4%) per 1,000 participants. FIT(47,47) participants had in the second round a higher positivity rate of 4.3%, a higher PPV for AN of 41.2% (95% CI: 40.2–42.1%), and a higher detection rate of AN of 15.0 (95% CI: 14.6–15.5%) per 1,000 participants. The SRR presented a significantly higher age‐adjusted positivity rate (SRR: 1.3, 95% CI: 1.1–1.5%) and age‐adjusted detection rate of AN (SRR: 1.5, 95% CI: 1.2–1.9%) in FIT(47,47) participants of the second round (Table 2). Differences in age‐adjusted PPV between the used cut‐offs were non‐significant.

The FIT(np,47) participants of the second round showed a high positivity rate of 7.9%, PPV of 54.9% (95% CI: 52.2–57.6%) for AN and detection rate of AN of 30.1 (95% CI: 27.9–32.3%) per 1,000 participants (Table 2). All outcomes were significantly higher compared to second‐round participants that had participated in the first round.

### Cumulative rates and NNScope

Of FIT(15,47) participants, 13.1% (95% CI: 12.8–13.4%) tested positive in the first (10.1%) or second (3.0%) round (cumulative positivity rate). This cumulative positivity rate was higher compared to the FIT(47,47) participants, of which 10.4% (95% CI: 10.3–10.5%) tested positive in the first (6.3%) or second (4.1%) round. The age‐adjusted cumulative positivity rate over two rounds was significantly higher in FIT(15,47) participants (SRR: 1.2 [1.2–1.3]).

**Figure 2:**
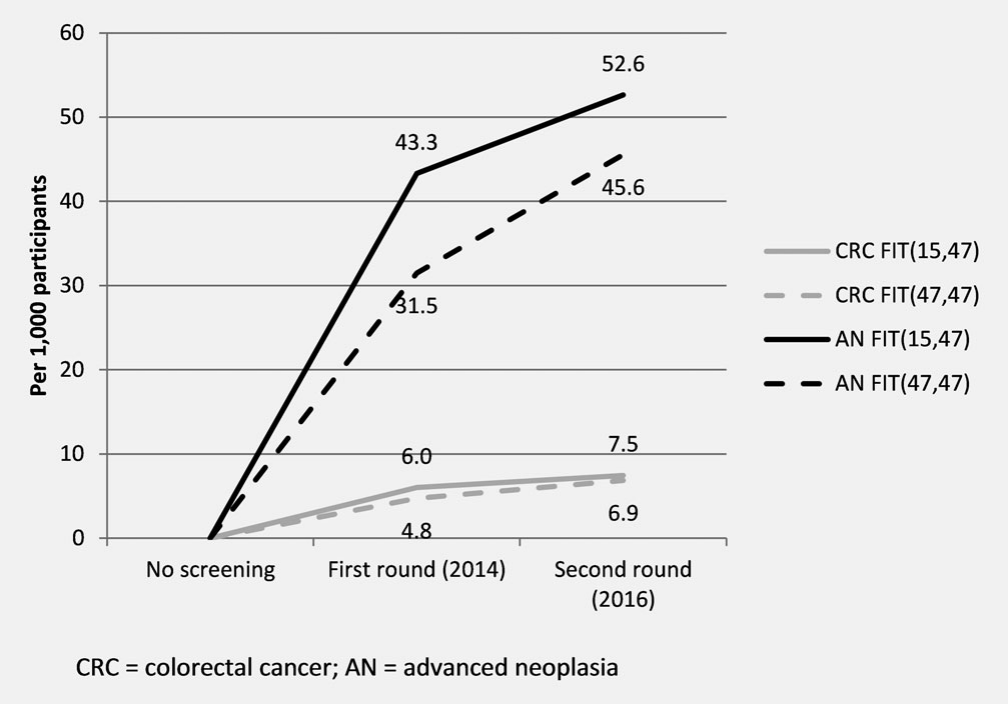
Cumulative detection rate of colorectal cancer and advanced neoplasia over two rounds of screening.

Per 1,000 FIT(15,47) participants, CRC was detected in 7.5 (95% CI: 6.7–8.3%) participants in the first (6.0) or second (1.4) round and AN were detected in 52.6 (95% CI: 50.6–54.8%) participants in the first (43.3) or second (9.3) round (cumulative detection rate; Fig. 2). A lower cumulative detection rate of CRC and AN was observed per 1,000 FIT(47,47) participants, in which 6.9 (95% CI: 6.6–7.2%) participants were detected with CRC in the first (4.8) or second (2.1) round and 45.6 (95% CI: 44.8–46.3%) with AN in the first (31.5) or second (14.1) round. Age‐adjusted rates showed a non‐significant difference in the cumulative detection rate over two rounds for CRC (SRR: 1.1, 95% CI: 0.8–1.4%), yet significantly more FIT(15,47) participants were detected with AN (SRR: 1.2, 95% CI: 1.1–1.3%).

**Figure 3:**
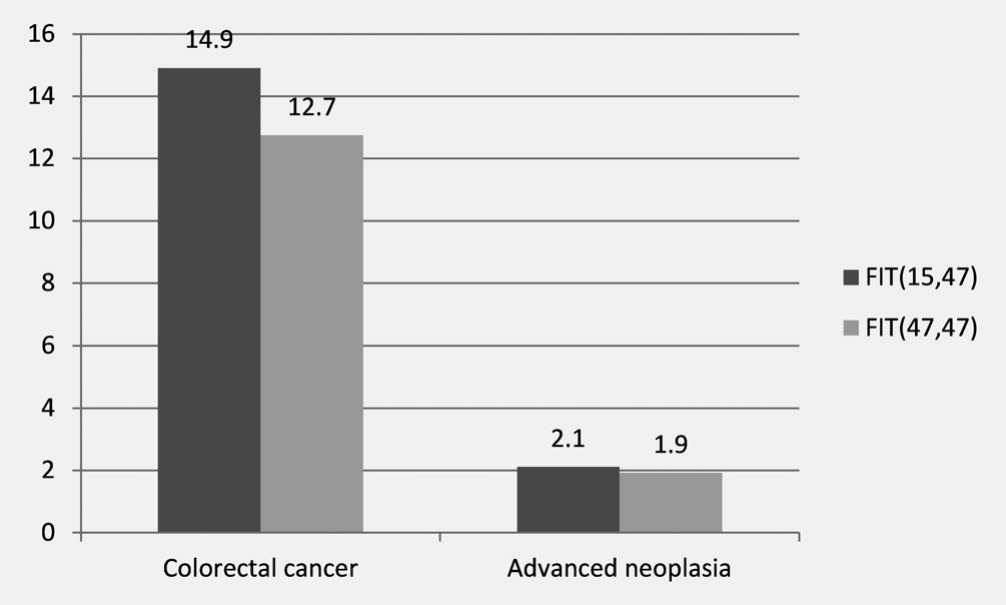
Number of participants that underwent colonoscopy (number needed to scope) over two rounds of screening to detect one colorectal cancer or advanced neoplasia.

Over two rounds, 14.9 (95% CI: 14.5–15.3%) FIT(15,47) participants and 12.7 (95% CI: 12.6–12.9%) FIT(47,47) participants needed to undergo colonoscopy (*NNScope*) to detect CRC in one participant, with a significant difference after age‐adjustment (SRR: 1.2, 95% CI: 1.1–1.3%). A similar pattern was observed for the NNScope to detect AN in one participant (Fig. 3).

### Yield by previous FIT result

The positivity rate, PPV and detection rate in the second round was strongly related to the concentration of Hb detected in feces in the first round. Participants with a FIT‐level in the first round between 15 and 47 μg Hb/g feces showed a positivity rate of 23.3%, a PPV for AN of 60.3% and a detection rate of AN of 120.3 per 1,000 participants in the second round (Table 3). The second round outcomes in these participants were significantly higher than in participants with a FIT‐level below 15 μg in the first round. Compared to participants with no (0 μg) detectable Hb in their feces sample in the first round, a participant with a FIT‐level between 15 and 47 μg was remarkably more likely to test FIT‐positive (OR 11.9, 95% CI: 11.3–12.5%) or have an AN detected during colonoscopy (OR 23.2, 95% CI: 21.5–25.1%). While the sensitivity analyses ruled out selection bias, it pointed out significant differences between male and female participants (Supporting Information Tables S1 and S2). However, this did not change the conclusion, as both genders presented a strong correlation between first round FIT‐result and outcomes in the subsequent round.

**Table 3:** Yield of the second round relative to FIT results of the first round

First screening round FIT result (Hb/g feces)	0 μg	>0 and <15 μg	≥15 and <47 μg	*p*‐value
Total	248,310	66,030	10,961	
Positivity rate				
*n* (%)	6,000 (2.4)	5,187 (7.9)	2,553 (23.3)	<0.001
Odds‐ratio (95% CI)	–	3.4 (3.3–3.5)^1^	11.9 (11.3–12.5)^1^	
PPV AN				
*n* (%)	1,390 (27.9)	1,993 (45.7)	1,319 (60.3)	<0.001
Odds‐ratio (95% CI)	–	2.2 (2.0–2.4)	3.9 (3.5–4.3)	
Detection rate AN				
*n* (per 1,000 participants)	1,390 (5.6)	1,993 (30.2)	1,319 (120.3)	<0.001
Odds‐ratio (95% CI)	–	5.4 (5.0–5.8)^1^	23.2 (21.5–25.1)^1^	

Note: Odds‐ratio are adjusted for age and gender.

1 Significant interaction between male and female gender (see Supporting Information).

Abbreviations: AN, advanced neoplasm; FIT, fecal immunochemical test; PPV, positive predictive value.

## Discussion

This study presents the results of the second round of the Dutch FIT‐based CRC screening program and evaluates the impact of increasing the FIT cut‐off in the first round on the yield of the second round.

We observed a consistently high participation as almost all first‐round participants also participated in the second round. The detection rate of AN in the second round was significantly higher in FIT(47,47) participants compared to FIT(15,47) participants. Nevertheless, the cumulative detection rate of AN over two rounds was significantly lower in FIT(47,47) participants. We found a strong correlation between the concentration μg Hb/g feces in the first round and the detection of AN in the subsequent round.

The Dutch government was the first to change the FIT cut‐off in a running national CRC screening program. Therefore we are the only country yet in which evaluation of the program can demonstrate the impact of adjusting the FIT cut‐off on the yield at a population level. The main reason to increase the FIT cut‐off during the first round was to reduce colonoscopy demand and the proportion of false‐positive FIT‐results. As previously reported, this indeed successfully decreased the positivity rate and increased the PPV in first round FIT(47,47) participants, at the cost of a lower detection rate.^13^ We demonstrated in the current study that, cumulatively, the detection rate over two rounds was still lower in FIT(47,47) participants. However, we also observed that the difference in cumulative detection rate of AN between FIT(47,47) and FIT(15,47) participants decreased from 11.8 AN per 1,000 participants in the first round, to 7.0 AN per 1,000 participants in the second round. This means that a substantial part of the missed lesions in the first screening‐round due to the increased FIT cut‐off from 15 to 47 μg Hb/g feces are detected at the subsequent round. The cumulative detection rate of CRC over two rounds in FIT(47,47) and FIT(15,47) participants was almost similar. If the difference in cumulative detection rate of AN between FIT(47,47) and FIT(15,47) participants keeps declining per subsequent screening, the impact of adjusting the FIT cut‐off on the cumulative detection rate might become insignificant within one or two subsequent screening rounds. However, it is unclear if the higher cut‐off resulted in more interval cancers and if the detected CRCs in the second round were still diagnosed in an early stage, which is a prerequisite for screening to be effective. Therefore, interval cancers and data on stage distribution have to be evaluated, before making final conclusions in this respect. Unfortunately, these data are not available yet.

The effect of increasing the cut‐off in the older birth cohorts, 75 and 76 years of age, which were not re‐invited for a second round as they exceeded the target age, should be assessed separately. As the missed lesions in FIT(47,47) participants of these birth cohorts will not be detected in a subsequent round, they might potentially progress to symptomatic CRC.

The reported participation rate (75.9%) in the Netherlands can be considered the highest in a subsequent round, even higher than seen in the Dutch CRC screening pilots.^17^, ^18^, ^19^ However, this outcome is overestimated by approximately 2%, since the calculation of participation rate excluded invitees who deregistered permanently during the first round and were therefore not invited to the second round. The favorable participation rate has been attributed to the non‐invasive screening modality and organizational structure of the program. The consistent high participation rate and the resulting detection rates of AA make it likely that the modeled long‐term effects in reducing CRC related mortality will be achieved.^10^ The consistent participation also allowed for missed findings in the first round due to the increased cut‐off to be detected in the second round. For FIT‐based CRC screening programs with a lower repeated participation rate, increasing the FIT cut‐off might have a larger impact.

The strong correlation between the level of Hb concentration of a negative FIT result and the chance of detecting AN in the subsequent round suggests an excellent opportunity for more personalized FIT screening based on Hb concentration. For example, the screening interval or FIT cut‐off of participants with no detectable blood (0 μg Hb/g feces) could potentially be increased. On the other hand, participants with a FIT result just below the cut‐off level might profit from more intense screening by shortening the screening interval. In concordance with our data, a similar association between FIT level and outcomes in the subsequent rounds has previously been presented by a Dutch, Spanish and Taiwanese study.^20^, ^21^, ^22^

Important strengths of our study are the nation‐wide implementation of the screening program, the large sample size and the well‐developed registration, therefore providing accurate data. Nevertheless, three limitations are noteworthy. As mentioned before, data on stage distribution are lacking, which would provide important information on the potential consequences of missed lesions in the first round caused by the increased FIT cut‐off. These data will be available in the near future. Second, for a final verdict on the consistency of the participation and the cumulative outcomes, information on more consecutive rounds is needed. Finally, not every birth cohort has been invited yet for the Dutch CRC screening program, hence conclusions are mainly based on a few age groups.

Notwithstanding these limitations, we are the first to present on how using a different FIT cut‐off in the first round impacts the outcomes of a subsequent round. Our findings are of value to other FIT‐based CRC screening programs considering an appropriate cut‐off in their setting, in particular when the used FIT cut‐off is within the same range.

In conclusion, participation in the Dutch CRC screening program was high and consistent. Our results show that using a higher FIT cut‐off in CRC screening has limited impact on the yield of screening because a substantial part of AN will be detected in subsequent rounds. To confirm whether these AN are still detected in an early stage, retrieving more information on the stage distribution of CRCs detected in the second round is important.

## Conflict of interest

E.D.: I have endoscopic equipment on loan of FujiFilm and received a research grant from FujiFilm. I have received an honorarium for consultancy from FujiFilm, Tillots and Olympus and a speaker's fee from Olympus and Roche. Besides, I am in the supervisory board of eNose. All other authors declare no conflict of interest.

## Supporting information


**Table S1**. FIT positivity rate and detection rate for AN in the second screening round relative to the first screening round FIT result, per gender
**Table S2**. Yield of the second round relative to FIT results of the first round, including only participants tested in the first round with 47 μg Hb/g feces

## Data Availability

The data that support the findings of this study are available from the corresponding author upon reasonable request.
