# Supplementary material for: The second round of the Dutch colorectal cancer screening program: Impact of an increased fecal immunochemical test cut‐off level on yield of screening
Source: Int J Cancer. 2020 Jan 9;147(4):1098–106. doi: 10.1002/ijc.32839 (PMC7383838; doi:10.1002/ijc.32839)
Supplement: Supplementary file 1 — Table S1. FIT positivity rate and detection rate for AN in the second screening round relative to the first screening round FIT result, per gender Table S2. Yield of the second round relative to FIT results of the first round, including only participants tested in the first round with 47 μg Hb/g feces [file IJC-147-1098-s001.docx]

**THE SECOND ROUND OF THE DUTCH COLORECTAL CANCER SCREENING PROGRAM:
IMPACT OF AN INCREASED FIT CUT-OFF LEVEL ON YIELD OF SCREENING**

A.I. Kooyker, BSc, E. Toes-Zoutendijk, MSc, A.W.J. Opstal-van Winden, PhD, M.C.W. Spaander, MD, PhD, M. Buskermolen, MD, A.J. van Vuuren, PhD, E.J. Kuipers, MD, PhD, F.J. van Kemenade, MD, PhD, C. Ramakers, PhD, M.G.J. Thomeer, MD, PhD, E. Dekker, MD, PhD, I. Nagtegaal, MD, PhD, H.J. de Koning, MD, PhD, M.E. van Leerdam, MD, PhD, MSc, I. Lansdorp-Vogelaar, PhD

Contents:

- Appendix 1: FIT positivity rate and detection rate for AN in the second screening round relative to the first screening round FIT result, per gender
- Appendix 2: Yield of the second round relative to FIT results of the first round, including only participants tested in the first round with 47 μg Hb/g feces

**Appendix 1**

| FIT positivity rate and detection rate for AN in the second screening round relative to the first screening round FIT result, per gender | | | |
| --- | --- | --- | --- |
| **First screening round FIT result (Hb/g feces)** | **0 µg** | **>0 µg and <15 µg** | **≥15 µg and <47 µg** |
| Positivity rate (OR (95% CI)) |  |  |  |
| Male | - | 3.2 (3.0 - 3.4) | 11.3 (10.5 - 12.0) |
| Female | - | 3.6 (3.4 - 3.8) | 12.8 (11.8 - 13.9) |
|  |  |  |  |
| Detection rate AN (OR (95% CI)) |  |  |  |
| Male | - | 5.0 (4.6 - 5.4) | 20.6 (18.6 - 22.7) |
| Female | - | 6.0 (5.4 - 6.7) | 28.2 (24.8 - 31.9) |
| Note: other outcomes were not significantly different between genders OR = Odds Ratio; AN = Advanced neoplasia | | | |

**Appendix 2**

| Yield of the second round relative to FIT results of the first round, including only participants tested in the first round with 47 μg Hb/g feces | | | | |
| --- | --- | --- | --- | --- |
| **First screening round FIT result (Hb/g feces)** | **0 µg** | **>0 µg and <15 µg** | **≥15 µg and <47 µg** | **P-value** |
| Total | 217435 | 57662 | 10948 |  |
| Positivity rate |  |  |  |  |
| n (%) | 5334 (2.5) | 4553 (7.9) | 2550 (23.3) | < 0.001 |
| Odds-ratio (95% CI) | - | 3.4 (3.2 - 3.5)* | 11.7 (11.1 - 12.3)* |  |
| PPV AN |  |  |  |  |
| n (%) | 1230 (27.8) | 1748 (45.7) | 1317 (60.3) | < 0.001 |
| Odds-ratio (95% CI) | - | 2.2 (2.0 - 2.4) | 3.9 (3.5 - 4.3) |  |
| Detection rate AN |  |  |  |  |
| n (per 1,000 participants) | 1230 (5.7) | 1748 (30.3) | 1317 (120.3) | < 0.001 |
| Odds-ratio (95% CI) | - | 5.4 (5.0 - 5.8)* | 22.9 (21.2 - 24.9)* |  |
| Note: Odds-ratio are adjusted for age and gender PPV = Positive Predictive Value; AN = Advanced Neoplasm | | | | |
